# The flagellin-TLR5-Nox4 axis promotes the migration of smooth muscle cells in atherosclerosis

**DOI:** 10.1038/s12276-019-0275-6

**Published:** 2019-07-10

**Authors:** Jinoh Kim, Jung-Yeon Yoo, Jung Min Suh, Sujin Park, Dongmin Kang, Hanjoong Jo, Yun Soo Bae

**Affiliations:** 1 Department of Life Science, Ewha Womans University, Seoul, Korea; 2 Department of Biotechnology, Emory University, Atlanta, GA USA

**Keywords:** Chemokines, RHO signalling

## Abstract

We hypothesized that NADPH oxidase 4 (Nox4) is involved in the formation of neointimal atherosclerotic plaques through the migration of smooth muscle cells (SMCs) in response to flagellin. Here, we demonstrate that TLR5-mediated Nox4 activation regulates the migration of SMCs, leading to neointimal plaque formation in atherosclerosis. To investigate the molecular mechanism by which the TLR5-Nox4 cascade mediates SMC migration, we analyzed the signaling cascade in primary vascular SMCs (VSMCs) from wild-type (WT) or Nox4 KO mice. Stimulation of VSMCs from Nox4 KO mice with flagellin failed to induce H_2_O_2_ production and Rac activation compared with stimulation of VSMCs from WT mice. Moreover, the migration of Nox4-deficient VSMCs was attenuated in response to flagellin in transwell migration and wound healing assays. Finally, we performed partial carotid artery ligation in ApoE KO and Nox4ApoE DKO mice fed a high-fat diet (HFD) with or without recombinant FliC (rFliC) injection. Injection of rFliC into ApoE KO mice fed a HFD resulted in significantly increased SMC migration into the intimal layer, whereas SMC accumulation was not detected in Nox4ApoE DKO mice. We conclude that activation of the TLR5-Nox4 cascade plays an important role in the formation of neointimal atherosclerotic plaques.

## Atherosclerosis: halting plaque in its tracks

TLR5-Nox4 signaling cascade plays a key role in atherosclerosis, hardening of the arteries, in which fatty deposits known as plaques accumulate in the inner walls of arteries. During plaque formation, vascular smooth muscle cells (VSMCs) migrate to the area and then collect fat molecules. The thickening plaques restrict blood flow. Inflammation was known to play a role in this process, but the activities of individual inflammatory molecules remained poorly understood. Yun Soo Bae at Ewha Womans University, Seoul, South Korea, and coworkers investigated how Nox4 affects plaque generation. They found that flagellin-TLR-5-Nox4 axis triggered VSMCs, leading to migrate and form an atherosclerotic plaque. In mice lacking Nox4, both VSMC migration and plaque formation were greatly reduced. These results may help in finding better treatments for this common cause of stroke and heart attack.

## Introduction

It has been well established that vascular inflammation plays an important role in the generation and progression of atherosclerosis^1–3^. Among various types of vascular inflammation, toll-like receptor (TLR)-mediated inflammation in vascular cells is known to be involved in atherosclerosis^4–7^. The expression of TLR1, TLR2, and TLR4 is enhanced in atherosclerotic lesions compared with healthy arteries. TLR isozymes in vascular cells, including endothelial cells (ECs), smooth muscle cells (SMCs), and macrophages, recognize endogenous and exogenous ligands and trigger the expression of downstream proinflammatory molecules, including monocyte chemotactic protein-1 (MCP-1), interleukin-6 (IL-6), interleukin-8 (IL-8), and metalloprotease (MMP), leading to atherosclerosis.

Several lines of evidence indicate that NADPH oxidase (Nox)-induced reactive oxygen species (ROS) generation serves as a second messenger in various cellular events^8,9^. To date, seven Nox isozymes (Nox 1–5 and Duox1–2) have been identified in different tissues and are activated by various agonists, resulting in the regulation of cell growth, differentiation and migration. We previously reported that Nox isozymes become linked with TLRs in ECs, SMCs, and macrophages in response to TLR agonist stimulation. Stimulation of ECs with lipopolysaccharide results in Nox4 activation through direct interaction with the COOH-terminus of TLR4, leading to the production of proinflammatory cytokines and adhesion molecules, including IL-8, MCP-1, and ICAM-1^10,11^. In the case of macrophages, TLR activates the Nox2 isozyme in a typical manner. Minimally oxidized low-density lipoprotein (mmLDL) is known to interact with CD14 and activate the TLR4 complex, inducing the secretion of certain cytokines in macrophages. MmLDL stimulates TLR4-mediated activation of Nox2 through a sequential signaling cascade involving Syk, phospholipase γ (PLCγ), and PKC, resulting in the production of proinflammatory cytokines, including IL-1β, IL-6, and RANTES^12,13^. The synthetic triacylated lipoprotein Pam3CSK4, a TLR2 agonist, stimulates H_2_O_2_ generation through interaction of Nox1 with TLR2 in mouse vascular smooth muscle cells (VSMCs)^14^. H_2_O_2_ generation by the TLR2-Nox1 axis plays a key role in the secretion of the proinflammatory cytokine MIP-2 in VSMCs. Secreted MIP-2 stimulates VSMC migration and vascular remodeling.

Recently, we reported that Nox4 activation by TLR5 stimulates inflammation of ECs and ultimately leads to atherogenesis^15^. Silencing of Nox4 expression by transfection of HAECs with Nox4-specific siRNA attenuated the expression levels of IL-8 and ICAM-1, leading to reduced adhesion and transendothelial migration of monocytes. Moreover, injection of recombinant FliC (rFliC) into Nox4ApoE DKO mice fed high-fat diets (HFDs) resulted in significantly decreased atherosclerotic plaque sizes compared with injection into ApoE KO mice, suggesting that Nox4 has proatherogenic activity in rFliC-mediated atherosclerosis. Moreover, injection of rFliC into ApoE KO mice increased the necrotic core size in the sinus valve due to infiltration of monocytes and neutrophils, accumulation of collagen and migration of VSMCs. In this report, we present the function of Nox4 in SMC migration for the generation of vulnerable plaques in response to FliC-TLR5 activation.

## Materials and methods

### Mice

All animal procedures were approved by the Institutional Animal Care and Use Committees (IACUC) at Ewha Womans University. Nox1 KO mice were purchased from The Jackson Laboratory (Bar Harbor, ME, USA). The generation of Nox4 KO mice has been described in a previous report^15^. Nox4 KO mice were crossed with ApoE KO mice to generate Nox4ApoE double knockout mice. rFliC purification and endotoxin elimination were performed as described in a previous report^15^. The animal protocols were in compliance with the NIH Guidelines for the Care and Use of Laboratory Animals and have been approved by the IACUC of the Center for Laboratory Animal Sciences, Ewha Industry-University Cooperation Foundation, Ewha Womans University.

### Preparation of murine vascular smooth muscle cells

VSMCs were prepared as described in a previous report^14^. VSMCs were isolated from the thoracic aortas of 8-week-old male wild-type, Nox1 knockout, or Nox4 knockout mice. Each mouse was killed by CO_2_ inhalation. An incision was made along the midline of the abdomen, the thorax was opened to expose the heart and lungs, and saline containing heparin was slowly perfused into the left ventricle of the heart. The adventitia and connective tissue that surround the aorta were carefully removed with fine forceps. The aorta was cut near the aortic arches, immersed in phosphate-buffered saline (PBS) containing 1% penicillin and streptomycin (Gibco Laboratories, Gaithersburg, MD, USA) and filled with collagenase type I (Worthington, Lakewood, NJ, USA; 268.32 U/mL, dissolved in serum-deprived Dulbecco’s modified Eagle’s medium(DMEM)) that had been sterilized by passage through a 0.2 μm Millipore membrane. After incubation at 80 rpm for 30 min at 37 °C, the aorta was chopped with a fine scissor and resuspended with collagenase type I solution. After incubation at 80 rpm for 90 min at 37 °C, the chopped aorta was centrifuged at 1000 rpm for 3 min, and the pellet was collected and resuspended in 20% FBS-DMEM. The cells were plated onto 35-mm-diameter culture dishes, and the dishes were placed in an incubator and left undisturbed for ~7 days. The purity of the VSMC preparations was confirmed with an antibody against smooth muscle actin (Supplementary Fig. 1). Three different batches of primary VSMCs were combined at passage 2, and the combined batch was used in all experiments at passages 4–7.

### Cell culture and antibodies for western blot analysis

The VSMCs were cultured at 37 °C in DMEM supplemented with 10% (v/v) fetal bovine serum and 1% (v/v) antibiotic-antimycotic solution (Gibco Laboratories, Gaithersburg, MD, USA). Cell lysates were subjected to SDS-PAGE and transferred to nitrocellulose membranes. The membranes were immunoblotted with anti-phospho-IκBα (Cell Signaling, Danvers, MA, USA, #2859), anti-IκBα (Cell Signaling, Danvers, MA, USA, #9242), anti-phospho-JNK (Cell Signaling, Danvers, MA, USA, #9251), or anti-JNK (Cell Signaling, Danvers, MA, USA, #9252) followed by horseradish peroxidase-conjugated secondary antibodies. The bands were visualized by chemiluminescence (Fujifilm LAS-3000).

### Partial carotid ligation surgery

All animal studies were carried out using 8-week-old male ApoE KO and Nox4ApoE DKO mice. All procedures were performed according to a protocol approved by the IACUC of Ewha Womans University. Partial ligation of the left carotid artery (LCA) was performed under anesthesia as previously described^16^ to induce low and disturbed flow in the LCA. All mice were fed a chow diet and given water ad libitum until partial ligation. Anesthesia was induced by inhalation of 2–2.5% (v/v) isoflurane. Epilated areas were disinfected with betadine, and a ventral midline incision (4–5 mm) was made in the neck. The LCA was exposed by blunt dissection. Three of four caudal branches of the LCA (the left external carotid, internal carotid, and occipital artery) were ligated with 6–0 silk sutures, while the superior thyroid artery was left intact. The incision was then closed with a 5–0 silk suture. The mice were monitored until recovery in a chamber on a heating pad after surgery. Following carotid ligation, disturbed flow in the LCA and laminar flow in the right carotid artery (RCA) was confirmed 1 day post ligation by ultrasonography using a VEVO 2100 system (VisualSonics, Toronto, Canada). The mice were fed Paigen’s high-fat diet (Research Diet, New Brunswick, NJ, USA) containing 1.25% cholesterol, 15% fat, and 0.5% cholic acid immediately following partial ligation for 10 days.

### Oil Red O staining

Ten days after the procedure, the carotid arteries were obtained and perfusion-fixed with 10% formalin. The samples were then embedded in OCT compound and frozen. Five frozen sections (each 6-µm thick) 1–2 mm proximal to the ligation site were obtained from each animal. The areas of the lumen, intima, and media were measured in sections stained with Oil red O for 10 min and analyzed with the ImageJ program. The mean value for five sections from each animal was used for analysis.

### Immunofluorescence staining

Frozen sections from carotid arteries were stained with anti-SMA (Santa Cruz Biotechnology, sc-6251, Dallas, TX, USA), anti-Flk-1 (Dako, M0851, Santa Clara, CA, USA), and anti-F4/80 (Abcam, ab6640, Cambridge, England) antibodies.

### rFliC purification and elimination of endotoxin

The preparation of rFliC has been described in a previous report^15^. Wild-type FliC (AA21–505) and a deletion mutant of FliC (AA119-505) from the *Salmonella enteritidis* FliC flagellin gene (GenBank Accession No. M84980) were cloned into pET15b vectors to generate His epitope-tagged recombinant proteins. The rFliC protein and mutant ΔFliC protein were eluted from Ni^+^-NTA resin (Qiagen, Hilden, Germany) with elution buffer containing 50 mM phosphate (pH 7.4), 20 mM NaCl, 100 mM imidazole and protease inhibitors. The rFliC and mutant ΔFliC proteins were passed through an EndoTrap Red endotoxin removal column (Hyglos) to remove potential endotoxin according to the manufacturer’s instructions. The endotoxin levels were measured using a Kinetic-QCL limulus amebocyte lysate (LAL) assay (Lonza). The LAL assays showed an endotoxin content of <0.1 EU/ml in the purified rFliC and mutant rFliC (ΔrFliC). Standard flagellin from *S. typhimurium* was purchased from InvivoGen. The flagellin used in this experiment contained 0.1–1 EU of endotoxin.

### H_2_O_2_ detection

Measurement of intracellular H_2_O_2_ was performed using DCF-DA and Peroxy Orange-1 (PO-1) as described in a previous report^15^.

### Transwell migration assay

Harvested wild-type and Nox4 knockout SMCs (8 × 10^3^ cells) were replated onto the upper chambers of Transwell filters with 8 μm pores (Corning, NY, USA) coated with 10 μg/mL fibronectin, and the chambers were placed in serum-free DMEM with or without 100 ng/mL ultrapure flagellin (InvivoGen, #tlrl-epstfla, San Diego, CA, USA). After 16 h, the cells were fixed with 70% methanol for 5 min. The cells on the underside of each filter were stained with hematoxylin for 5 min and rinsed with distilled water. The filter was stained with eosin for 3 min and washed again. Nonmigrating cells on the upper side of the filter were removed with cotton swabs. Images were captured using a Nikon Eclipse 80i microscope. Three independent filters were analyzed for each experiment by counting the number of cells on each filter in five random fields.

### Cell proliferation assay (MTT assay)

Wild-type (WT) and Nox4 KO SMCs (2 × 10^3^ cells/well) were seeded into 96-well plates and stimulated with flagellin (100 ng/mL) for 24 h. The cells were incubated for 4 h with MTT labeling reagent, and then solubilization solution was added (Cell Proliferation Kit I, Roche, Cat. No. 11 465 007 001, Basel, Switzland). The absorbance was measured at 590 nm.

### Wound healing assay

SMCs (1 × 10^5^ cells) were plated onto 6-well plates. After the cells reached 90% confluence, the cells were serum-starved for 4 h and then treated with mitomycin (5 μg/mL) for 1 h. The cells were scratched with 200 μL pipette tips, and the starting point was marked with a marker pen at the bottom of the plate. The cells were washed with PBS three times, the medium was replaced with 0.5% FBS medium containing flagellin (100 ng/mL), and the cells were incubated for 24 h. Images were captured using an Axiovert 40 C inverted microscope (Carl Zeiss, Oberkochen, Germany) equipped with a PowerShot A640 digital camera (Canon, Tokyo, Japan).

### Single-cell tracking through confocal microscopy^17^

SMCs were prepared for live-cell imaging prior to being placed in an incubation chamber (Chamlide TC; Live Cell Instrument, Seoul, Korea) kept at 37 °C and 5% CO_2_. Briefly, the cells were cultured in a 12-well dish containing coverslips (diameter = 12 mm, Marienfeld, Germany) coated with 0.01% poly-L-lysine (Sigma-Aldrich, St. Louis, MO, USA). The cells were stained with 5 μM DRAQ5 (1,5-bis{[2-(di-methylamino) ethyl] amino}-4,8-dihydroxyanthracene-9,10-dione, ab108410, Abcam, Cambridge, UK), a cell permeable far-red fluorescent DNA dye, to track single-cell migration. At the same time, the cells were stimulated with or without flagellin (100 ng/mL). Confocal images were obtained with a Nikon A1R confocal microscope for 4 h using a 60× Plan Apochromat VC objective (NA 1.40) under illumination with a 633 nm laser. All images were processed, and quantification for single-cell tracking was performed using Nikon NIS Element C software (version 3.10).

### Quantitative real-time PCR analysis

Quantitative real-time PCR was used to analyze the expression levels of Nox isozymes using a TaqMan gene expression assay (Applied Biosystems, Foster City, CA, USA). The experiments were performed with KAPA PROBE FAST Universal 2× qPCR Master Mix (KAPA Biosystems, Basel, Switzerland). Glyceraldehyde-3-phosphate dehydrogenase (*GAPDH*) served as a housekeeping gene and internal control. The expression levels of other genes were quantified with a KAPA SYBR^®^ FAST qPCR Kit (KAPA Biosystems, Basel, Switzerland) using the following specific primers: mouse ICAM-1, 5’-AGGTGGTTCTTCTGAGCGGC-3’ and 5’-AAACAGGAACTTTCCCGCCA-3’; mouse VCAM-1, 5’-TCTTGGGAGCCTCAACGGTA-3’ and 5’-CAAGTGAGGGCCATGGAGTC-3’; mouse MCP-1, 5’-GAAGGAATGGGTCCAGACAT-3’ and 5’-ACGGGTCAACTTCACATTCA-3’; mouse RANTES, 5’- TGCCCACGTCAAGGAGTATTTC-3’ and 5’-AACCCACTTCTT CTCTGGGTTG-3’; mouse IL-1β, 5’-CAACCAACAAGTGATATTCTCCATG-3’ and 5’-GATCCACACTCTCCAGCTGCA-3’; mouse TNF-α, 5’-CACGTCGTAGCAAACCACCAAGTGGA-3’ and 5’-TGGGAGTAGACAAGGTACAACCC-3’; mouse IL-6, 5’-TCCGGAGAGGAGACTTCACA-3’ and 5’-TGCAAGTGCATCATCGTTGT-3’; and mouse 18S, 5’-AGGAATTGACGGAAGGGCACCA-3’ and 5’-GTGCAGCCCCGGACATCTAAG-3’. The 18S ribosomal RNA gene served as a housekeeping control. The fold changes in target gene expression were normalized by analyzing the cycle number for each gene with the 2^−ΔΔCt^ method.

### Determination of IL-6 protein levels

SMCs (1 × 10^5^ cells) from WT or Nox4 KO mice were incubated with or without flagellin (100 ng/mL) for 12 h, and then the medium was collected. The IL-6 protein levels were determined with an ELISA kit according to the manufacturer’s instructions (R&D Systems, Minneapolis, MN, USA, M6000B).

### Rac activity assay

Glutathione Sepharose 4B-conjugated GST-PAK-RBD was prepared as previously described for a Rac activity assay^18^. The bead-conjugated GST–PAK–RBD was incubated for 1 h at 4 °C in lysis buffer with lysates of wild-type and Nox4 knockout SMCs. The beads were then separated by centrifugation, washed three times and subjected to immunoblot analysis with monoclonal antibodies against Rac1 (Millipore, Billerica, MA, USA).

### RhoA activity assay

RhoA activity was measured using a RhoA pulldown assay Kit (Cytoskeleton, Denver, CO, USA) according to the manufacturer’s instructions. Briefly, WT and Nox4 KO SMCs were serum-starved for 16 h and stimulated with flagellin (100 ng/mL) for 1 h. The cells were lysed on ice in lysis buffer and then centrifuged at 12,000 × *g* for 5 min. The cell debris was removed, and the supernatants were incubated with rhotekin-RBD beads for 1 h at 4 °C on a rotator. The beads were washed, and the immunoprecipitated complex was resuspended in 2× SDS sample buffer and subjected to 12% SDS-PAGE followed by western blotting.

### Statistical analysis

The data are given as the mean ± SD or the mean ± SEM. Pairwise comparisons were performed using Student’s *t*-tests. Multiple comparisons of means were calculated using one-way analysis of variance (ANOVA) (GraphPad Software, La Jolla, CA, USA). Differences between groups were considered significant at *P* values < 0.05.

## Results

### Nox4 is required for flagellin-induced H_2_O_2_ generation in smooth muscle cells

**Fig. 1:**
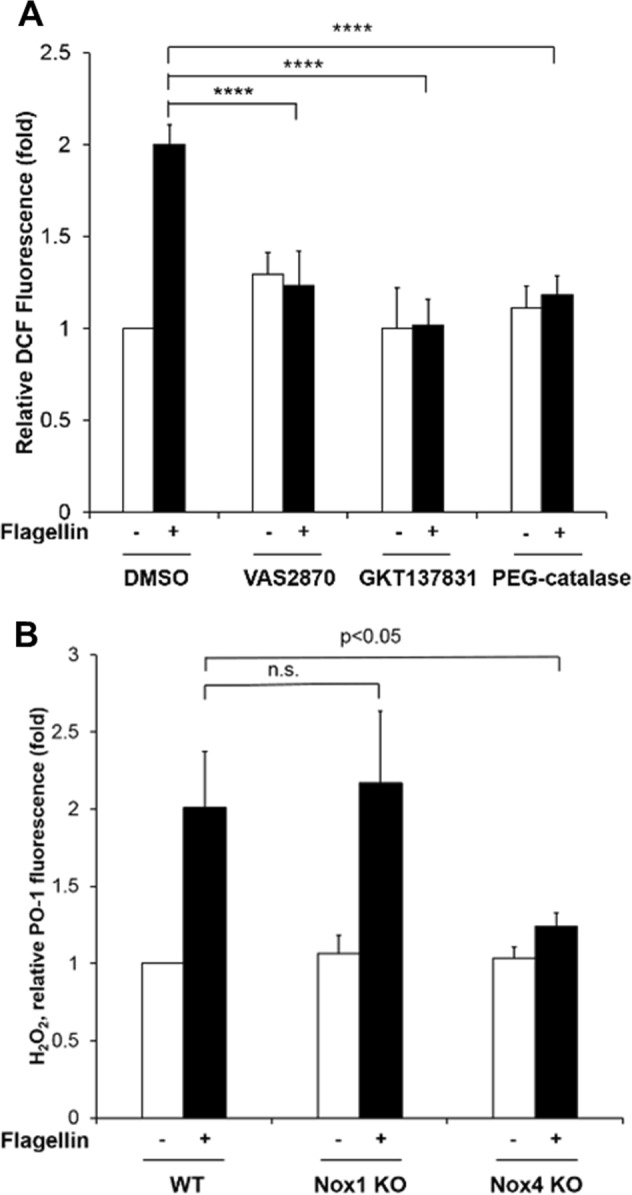
Nox4 is required for flagellin-induced H_2_O_2_ generation in smooth muscle cells. **a** WT SMCs were pretreated with VAS2870 (10 μM), GKT137831 (10 μM), or PEG-catalase (50 U/mL) for 30 min, stimulated with flagellin (100 ng/mL) for 10 min, and then incubated with DCF-DA for 10 min. The generation of ROS was monitored by confocal microscopic analysis of DCF fluorescence (*N* = 3, mean ± SD, ****p < 0.0005). **b** WT, Nox1 KO, and Nox4 KO mouse SMCs were serum-starved for 16 h and incubated for 15 min in the dark at 37 °C with 5 μM PO-1. Then, flagellin (100 ng/mL) was added to the dye/cell mixture. The generation of H_2_O_2_ was monitored by confocal microscopic analysis of PO-1 fluorescence (*N* = 3, mean ± SD, n.s. represents nonsignificance, *p < 0.05)

We previously reported that the flagellin-TLR5-Nox4 axis mediates inflammation processes in aortic ECs, leading to the formation of initial atherosclerotic plaques^15^. During the progression of atherosclerosis, atherosclerotic lesions became vulnerable plaques that contain large necrotic cores and exhibit vascular remodeling, including SMC migration and extracellular matrix (ECM) accumulation. To evaluate the function of Nox4 in the formation of atherosclerotic plaques, ROS generation was examined in primary mouse VSMCs (Supplementary Fig. S1) in response to flagellin by measuring the fluorescence of 2’,7’-DCF-DA by confocal microscopy. Stimulation of WT mouse SMCs with flagellin significantly increased ROS generation (Fig. 1a). Flagellin-induced ROS generation was inhibited by pretreatment with the Nox inhibitors VAS2870 and GKT137831 (Fig. 1b). To validate which ROS is involved in flagellin stimulation, we treated cells with cell-permeable polyethylene glycol (PEG)-conjugated catalase. PEG-conjugated catalase completely suppressed ROS generation, indicating that the predominant flagellin-dependent ROS is H_2_O_2_ (Fig. 1a). We wondered whether the Nox isozyme is responsible for H_2_O_2_ generation in response to flagellin. We first measured the levels of Nox isozyme expression in primary VSMCs. Nox4 was the predominant isozyme, and the Nox1 isozyme was the minor form in primary VSMCs (Supplementary Fig. S2). We prepared primary VSMCs from the aortas of WT, Nox4 KO and Nox1 KO mice and detected H_2_O_2_ generation by using a Peroxy Orange-1 (PO-1) dye, which is sensitive specifically to H_2_O_2_. Stimulation of WT and Nox1 KO VSMCs with flagellin resulted in significantly increased H_2_O_2_ generation, whereas Nox4 KO VSMCs failed to generate H_2_O_2_ in response to flagellin (Fig. 1b).

### Flagellin-dependent NF-kB activation requires Nox4 activity in SMCs

**Fig. 2:**
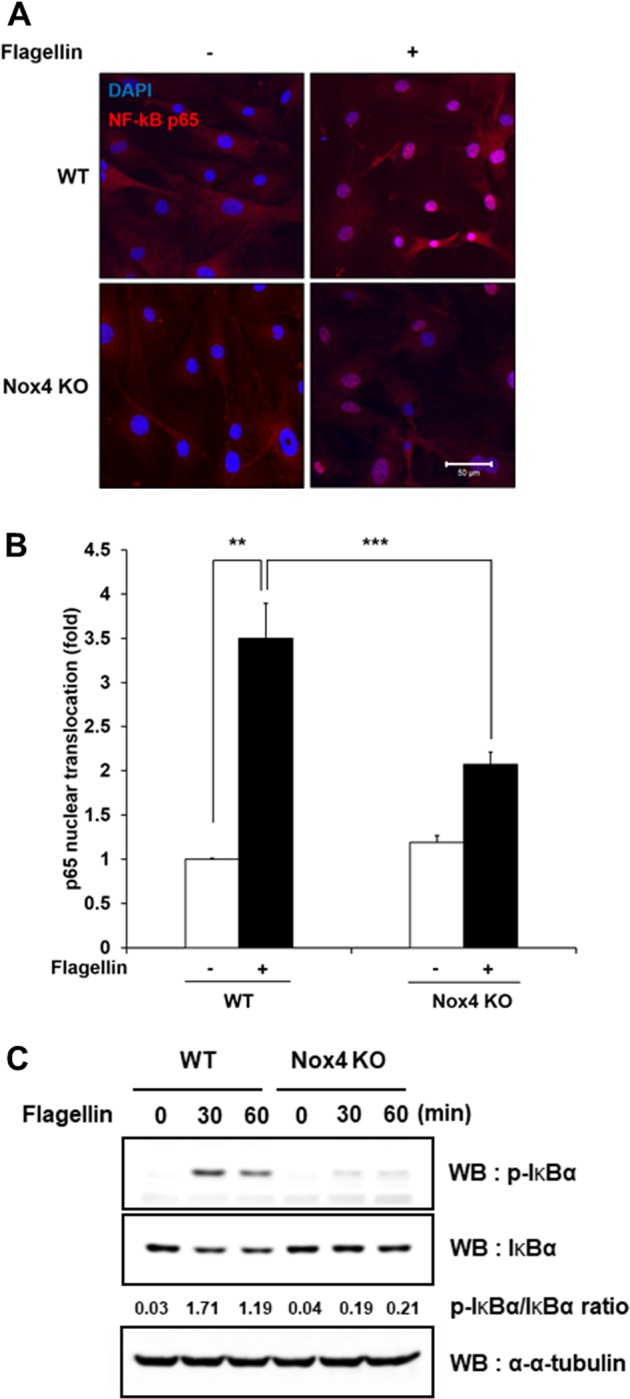
Nox4 is required for flagellin-induced NF-kB activation in smooth muscle cells. **a** WT and Nox4 KO SMCs were stimulated with flagellin (100 ng/mL) for 1 h and stained with a p65 antibody (red) and DAPI (blue). A representative image is shown. Scale bar = 50 μm. **b** Nuclear-translocated p65 was quantified using the ImageJ program (*N* = 3, mean ± SD, **p < 0.01, ***p < 0.001). **c** Immunoblotting of phosphorylated IkBα and IkBα

In a previous report, we showed that the TLR5-Nox4-H_2_O_2_ cascade induces the activation of NF-kB as a hallmark of inflammatory signaling in the endothelium^15^. We next validated whether the TLR5-Nox4-H_2_O_2_ axis regulates NF-kB activation in VSMCs. Stimulation of SMCs with flagellin failed to induce nuclear localization of RelA/p65, a subunit of NF-kB, in Nox4 KO SMCs compared with WT SMCs, indicating that the TLR5-Nox4 cascade stimulates NF-kB signaling (Fig. 2a, b). To confirm the flagellin-dependent NF-kB activation in VSMCs, we measured IkB phosphorylation and degradation in response to flagellin. Stimulation of WT SMCs with flagellin resulted in increased IkBα phosphorylation and degradation (Fig. 2c). Moreover, the ratio of phosphorylated IkBα to IkBα was significantly increased in WT SMCs but not in Nox4 SMCs (Fig. 2c). However, Nox4 KO SMCs failed to phosphorylate and degrade IkB in response to flagellin (Fig. 2c). These results show that flagellin stimulates NF-kB activation through IkB degradation. These results indicated that Nox4 is an essential molecule in flagellin-dependent NF-kB activation in SMCs.

### Function of the TLR5-Nox4-NF-kB axis in the flagellin-induced expression of proinflammatory molecules in SMCs

**Fig. 3:**
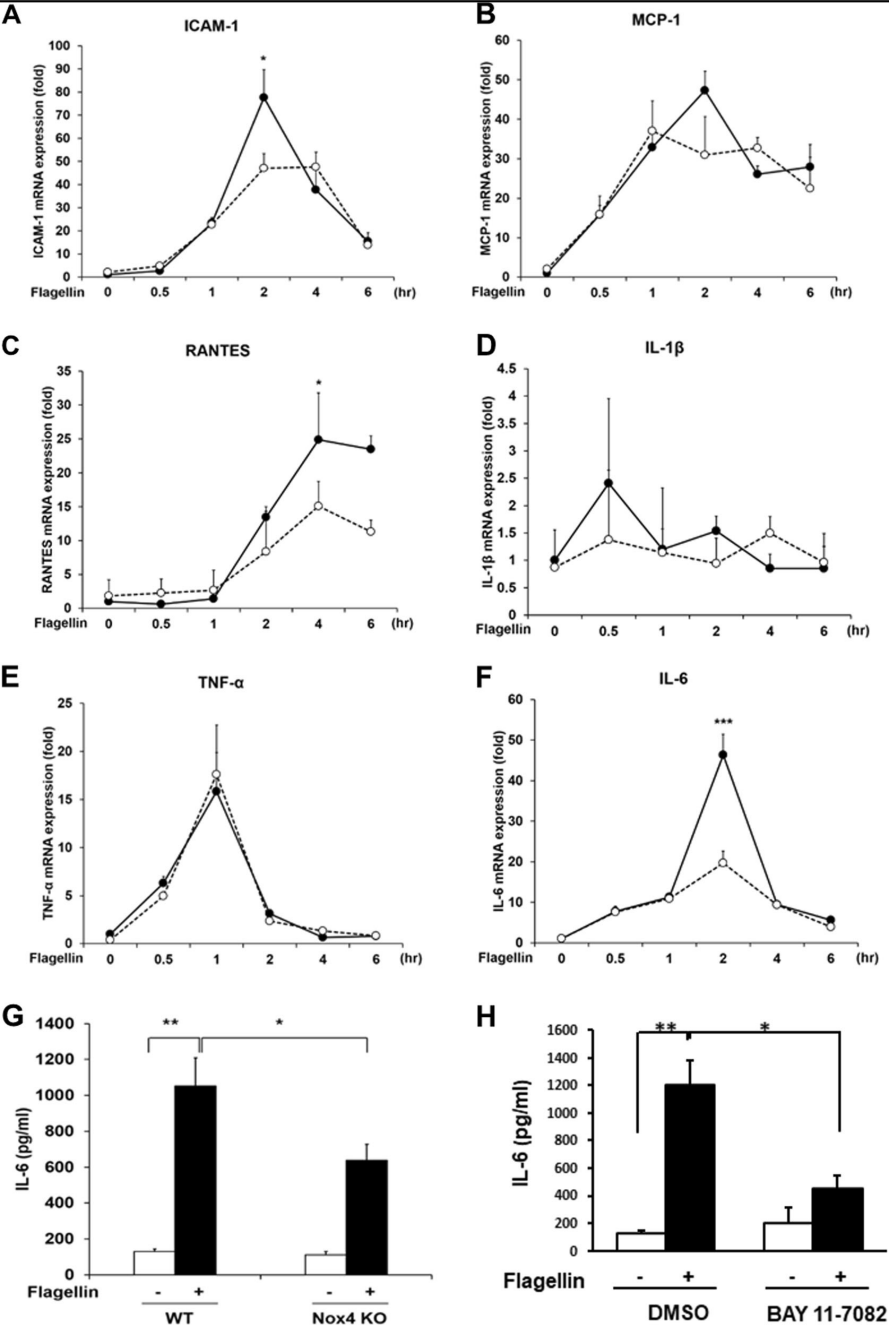
Nox4 is required for flagellin-induced inflammation in smooth muscle cells. The results are presented as fold increases in mRNA levels in flagellin-stimulated cells compared with unstimulated cells (black circles: WT SMCs, white circles: Nox4 KO SMCs) (**a**–**g**). **a** Quantification of ICAM-1 mRNA expression in VSMCs (*N* = 3, mean ± SD, compared with the Nox4 KO SMC group, *p < 0.05). **b** Quantification of MCP-1 mRNA expression. (*N* = 3, mean ± SD). **c** Quantification of RANTES mRNA expression (*N* = 3, mean ± SD, *p < 0.05). **d** Quantification of IL-1β mRNA expression (*N* = 3, mean ± SD). **e** Quantification of TNF-α mRNA expression (*N* = 3, mean ± SD). **f** Quantification of IL-6 mRNA expression (*N* = 3, mean ± SD, ***p < 0.001). **g** WT and Nox4 KO SMCs were stimulated with flagellin (100 ng/mL) for 12 h. IL-6 protein secretion was analyzed by ELISA (*N* = 3, mean ± SD, *p < 0.05, **p < 0.01). **h** Effect of an NF-kB inhibitor (BAY11-7082) on IL-6 expression in WT SMCs

We next explored whether the TLR5-Nox4 axis regulates the production of proinflammatory cytokines in SMCs. The differential expression of proinflammatory cytokines between WT and Nox4 KO SMCs was determined. The expression of the chemoattractants RANTES and IL-6 and the adhesion molecule ICAM-1 was downregulated in Nox4 KO SMCs in response to flagellin (Fig. 3a, c, and f). However, the expression profiles of MCP-1, IL-1β, and TNF-α were not changed in either group of cells in response to flagellin (Fig. 3b, d, and e). Moreover, we found that the protein levels of IL-6 were attenuated in flagellin-treated Nox4 KO SMCs compared with WT SMCs (Fig. 3g). Many reports have suggested that the flagellin-TLR5-Nox4-NFkB axis stimulates the expression of proinflammatory cytokines. We attempted to determine whether this signaling axis regulates IL-6. Pretreatment of SMCs with Bay 11–7082, an NF-kB inhibitor, resulted in suppressed IL-6 expression, indicating that NF-kB activation by the TLR5-Nox4 axis leads to the expression of proinflammatory cytokines, including IL-6 (Fig. 3h).

### The TLR5-Nox4 cascade regulates flagellin-induced smooth muscle cell migration

**Fig. 4:**
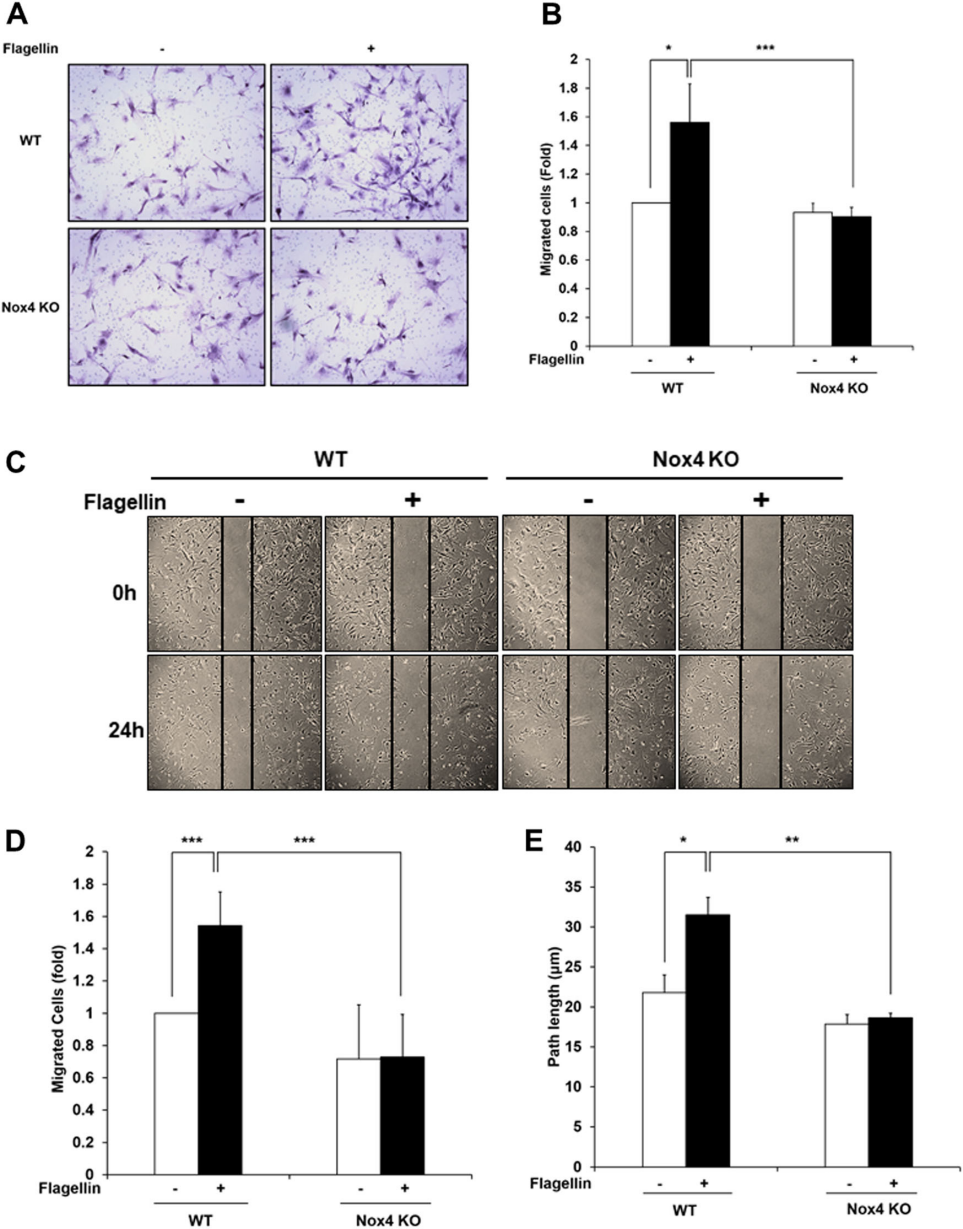
Nox4 regulates the flagellin-induced migration of smooth muscle cells. **a** Transwell migration assay. WT and Nox4 KO SMCs were seeded into the upper chamber, and stimulating medium containing flagellin (100 ng/mL) was added to the lower chamber. After 16 h, the migrated cells were stained with hematoxylin/eosin and counted. Representative images are shown for each group. **b** Quantification of the transwell migration assay results (*N* = 5, mean ± SD, *p < 0.05, ***p < 0.001). **c** Wound healing assay. After WT and Nox4 KO SMCs (initial cell number: 1 × 10^5^ cells) reached 90% confluence, the cells were serum-starved for 4 h and then pretreated with mitomycin C (5 μg/mL) for 1 h. The SMCs were subjected to injury by scratching and then incubated with or without flagellin (100 ng/mL) for 24 h. **d** The migrated cells in the wound healing assay were quantified (*N* = 5, mean ± SD, ***p < 0.001). **e** The path length of the migrating cells after 4 h is presented as the mean ± SD from three independent experiments (*n* = 9 cells). **p < 0.01 (Student’s *t-* test)

We then investigated whether a TLR5-Nox4-H_2_O_2_ cascade regulates SMC migration. Transwell migration assays with WT and Nox4 KO SMCs in the absence or presence of flagellin were performed. Migration of WT SMCs was enhanced in the presence of flagellin, whereas Nox4 KO SMCs failed to migrate in response to flagellin (Fig. 4a, b). It has been reported that phenotypic changes in SMCs contribute to cell migration as well as proliferation^19^. We next investigated whether Nox4 regulates the proliferation of SMCs. Stimulation of VSMCs from WT mice with flagellin resulted in significant increases in cell proliferation (Supplementary Fig. S3). However, flagellin failed to induce cell proliferation in VSMCs from Nox4 KO mice (Supplementary Fig. S3). These results are consistent with previous results^20–22^. To eliminate the effect of proliferation on VSMC migration, we pretreated cells with mitomycin C (5 μg/mL) for 1 h before flagellin stimulation in a migration assay. Mitomycin C is a well-known potent DNA crosslinker that prevents cell proliferation. To confirm the effect of Nox4 on SMC migration, we performed a wound healing assay with cells pretreated with mitomycin C. Flagellin stimulated WT SMC migration into the wound area (Fig. 4c, d). However, Nox4 KO SMCs did not efficiently migrate into the wound area (Fig. 4c, d). To validate the role of Nox4 in SMC migration in response to flagellin, we attempted live cell imaging with both groups of SMCs in the presence of flagellin. Live single-cell tracking analysis showed that the migration path length of Nox4 KO SMCs was reduced by 35% compared with that of WT SMCs in response to flagellin (Fig. 4e and Supplementary Fig. S4). These results demonstrated that Nox4 plays an important role in SMC migration.

### Flagellin-induced SMC migration requires RhoA and Rac1 activation through JNK activation

**Fig. 5:**
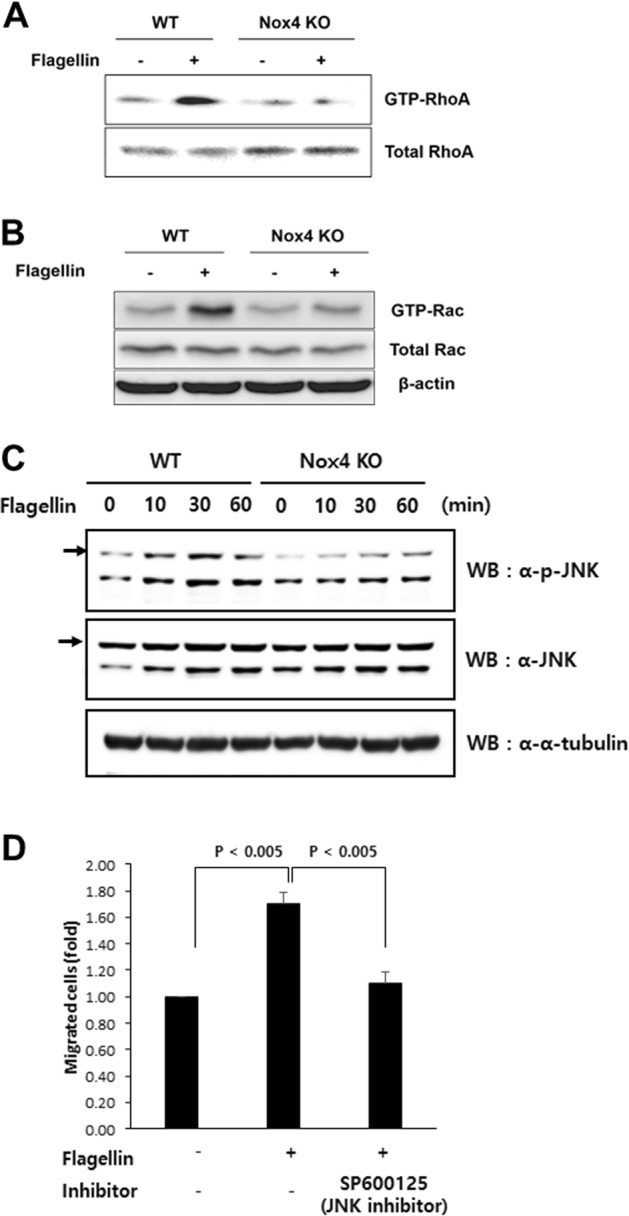
Deficiency of Nox4 decreased RhoA and Rac activation in response to flagellin. **a** RhoA activity assay. WT and Nox4 KO SMCs were stimulated with flagellin (100 ng/mL) for 60 min and subjected to a pulldown assay for RhoA activity. The amount of immunoprecipitated GTP-RhoA was analyzed by immunoblot analysis with anti-RhoA. Total cell lysates were analyzed for RhoA expression as a loading control. **b** Rac activity assay. WT and Nox4 SMCs were stimulated with flagellin (100 ng/mL) for 60 min. Cell lysates were incubated with bead-bound GST-PAK-RBD for 1 h. The bound proteins on the beads were subjected to immunoblot analysis with anti-Rac. Total cell lysates were analyzed for Rac expression as a loading control. **c** Immunoblot analysis of phosphorylated JNK in cells stimulated with flagellin (100 ng/mL) for the indicated times. **d** Effect of a JNK inhibitor (SP600125) on JNK phosphorylation in WT SMCs

We investigated the molecular mechanism by which the TLR5-Nox4 cascade regulates SMC migration. Rho family GTPases, such as RhoA and Rac1, are well-known molecules in cell migration. We investigated the activation of RhoA and Rac1 in SMCs in response to flagellin. Flagellin greatly enhanced the activation of RhoA and Rac1 in WT SMCs, whereas neither GTPase was activated in Nox4 KO SMCs in response to flagellin (Fig. 5a, b). Several lines of evidence suggest that the TLR5-mediated signaling cascade is involved in JNK activation^23–25^. We next investigated flagellin-mediated JNK activation in Nox4-deficient SMCs. Flagellin stimulated JNK activation in WT SMCs but failed to activate it in Nox4 KO SMCs (Fig. 5c). To validate the role of JNK in cell migration, SP600125, a JNK inhibitor, was applied, and flagellin-mediated cell migration was assessed. Treatment of SMCs with SP600125 suppressed cell migration, indicating that JNK activation is an essential event during flagellin-induced SMC migration (Fig. 5d).

### Reduced atherosclerotic lesion formation and SMC migration in Nox4 KO mice after partial carotid artery ligation

**Fig. 6:**
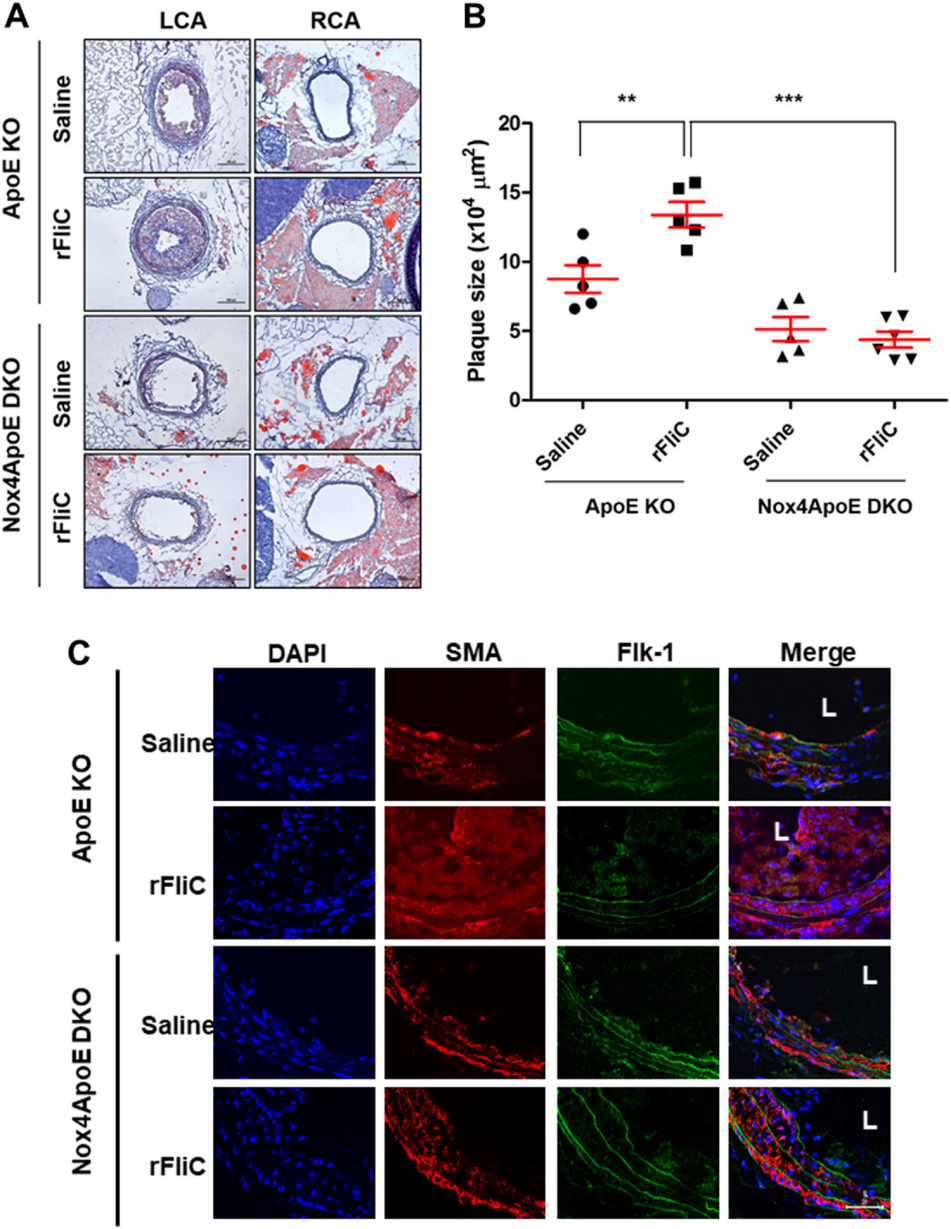
Nox4 regulates flagellin-induced plaque formation and SMC migration in a partial carotid artery ligation model. **a** Oil red O staining of the left carotid artery (LCA) and right carotid artery (RCA) 10 days after partial ligation surgery (1 mm from the ligation site, scale bar = 200 μm). **b** Quantification of plaque sizes using ImageJ (*N* = 5, mean ± SEM, **p < 0.01, ***p < 0.001). **c** Immunofluorescence for SMA (red) and Flk-1 (green). The color-merged images are shown in the right panels. Scale bar = 50 μm

To determine the effect of Nox4 on neointimal formation in arteries, we established a carotid artery injury model through partial carotid artery ligation in Nox4 KO mice. High-resolution ultrasound (VEVO 2100 high-resolution in vivo microimaging ultrasound system, VisualSonics) provided the flow velocity profiles of the mouse carotid arteries. Partial ligation of the left common carotid artery (LCA) induced flow reversal (as indicated by the arrows in Supplementary Fig. S5) during diastole, while flow in the right common carotid artery (RCA) remained unchanged after ligation of the LCA. We found that partial ligation of the LCA in WT mice induced atherosclerotic lesions and that injection of rFliC with partial ligation of the LCA accelerated atherosclerosis (Fig. 6a, b). However, the atherosclerotic lesions associated with partial ligation of the LCA or with injection of rFliC combined with partial ligation of the LCA were significantly suppressed in Nox4 KO mice. These results indicated that deficiency of Nox4 significantly inhibits the development of atherosclerosis induced by partial ligation and rFliC injection. To verify the migration of SMCs and the infiltration of macrophages in this animal model, we quantified the SMCs and macrophages in the intima in mice subjected to partial ligation of the LCA. SMC migration (Fig. 6c) and macrophage infiltration (Supplementary Fig. S6) were significantly increased in ApoE KO mice subjected to rFliC injection and partial ligation of the LCA, whereas migration and infiltration were not detected in Nox4ApoE DKO mice. These results strongly indicate that the flagellin-TLR5-Nox4 axis is essential for SMC migration and macrophage infiltration in the development of atherosclerosis.

## Discussion

**Fig. 7:**
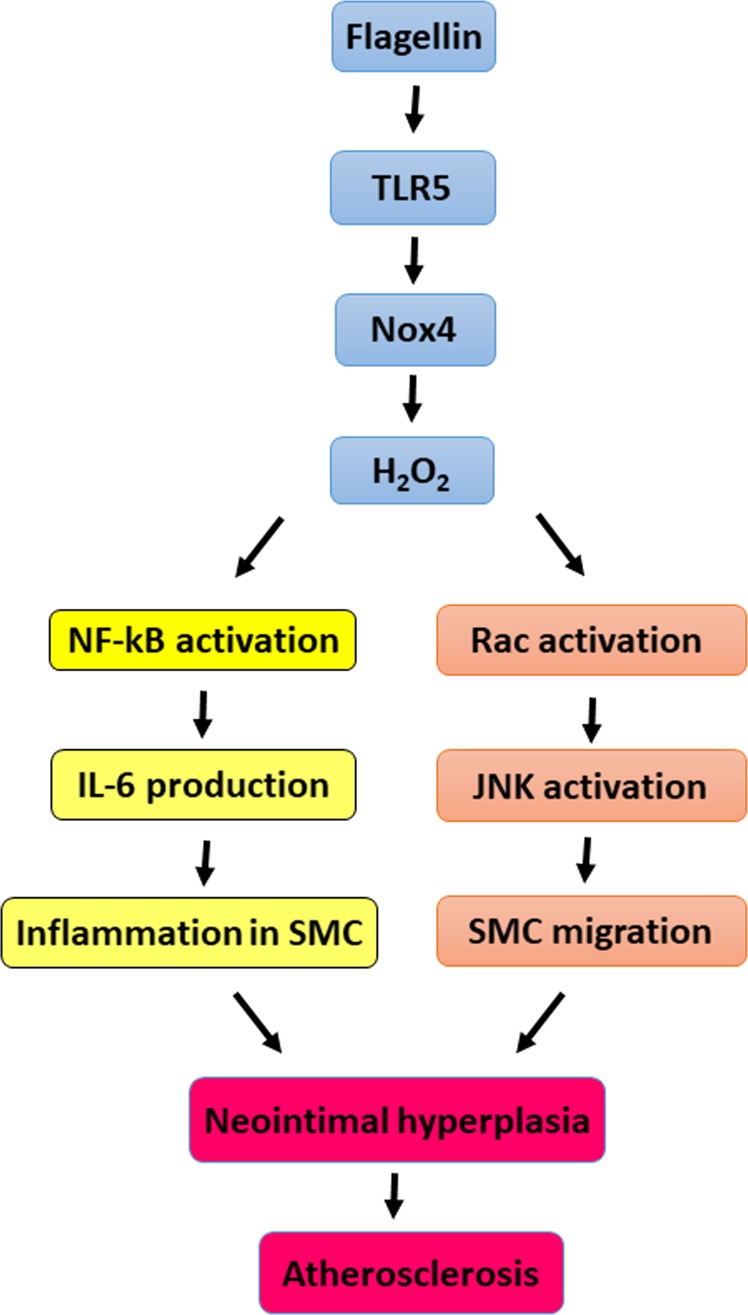
Proposed function of the TLR5-Nox4 cascade in the migration of smooth muscle cells. In SMCs, the flagellin-TLR5-Nox4 axis stimulates H_2_O_2_ generation. Nox-mediated H_2_O_2_ production induces NF-kB activation, leading to IL-6 production for inflammation of SMCs and activating the Rac-JNK cascade for SMC migration. We have demonstrated that the flagellin-TLR5-Nox4 cascade is an important mediator in the formation of neointimal hyperplasia in atherosclerosis

The formation of initial atherosclerotic plaques is induced by monocyte/macrophage interaction with the endothelium, monocyte/macrophage infiltration, and foam cell formation, which leads to neointimal hyperplasia. Next, lesion progression includes SMC migration into the neointimal area^1–3^. The pathological intimal thickening in mid-stage atherosclerosis is tightly associated with uptake of modified lipoproteins by VSMCs, apoptosis of VSMCs, and accumulation of extracellular protective fibrous caps overlying the thrombotic lipid cores of advanced lesions^26,27^. We previously revealed the role of the TLR5-Nox4 axis in atherogenesis in response to flagellin stimulation^15^. We demonstrated that the TLR5-Nox4 cascade, in particular, induces the proinflammatory stage in ECs, leading to the formation of atherosclerotic plaques. In this study, we found that Nox4 deficiency attenuated the expression of proinflammatory cytokines in SMCs and the migration of SMCs in response to flagellin stimulation, indicating that the flagellin-TLR5-Nox4 signaling cascade is an important mediator in the formation of intimal hyperplasia in atherosclerosis (Fig. 7).

When different types of cells are stimulated by specific agonists, each cell type shows a distinct and specific response. This likely involves specific responses by cell surface receptors as well as cell type-specific communication between downstream signaling proteins. Many studies have reported that flagellin stimulates proinflammatory cytokine production in various cell types^28–31^. We have previously shown that the flagellin-TLR5-Nox4 axis stimulates IL-8 expression in ECs. However, we present here that the flagellin-Nox4 cascade induces IL-6 expression in SMCs. Flagellin-mediated IL-1β or TNF-α production in gastric mucosal cells and hepatocytes is connected with cellular transformation^20–22^. Based on previous reports and our results, we can assume that flagellin stimulation in vascular cells such as ECs and SMCs regulates IL-8 or IL-6 expression, while in mucosal epithelial cells, the agonist induces IL-1β or TNF-α production. NF-kB activation plays an important role in the production of proinflammatory cytokines, including IL-1β, TNF-α, IL-8, and IL-6. Flagellin activates NF-kB as well as MAPK and PKA^30,32–34^. Various cytosolic kinases and their signaling cascades communicate with each other, leading to downstream events. For example, MAPK stimulates NF-kB transcriptional activity, whereas PKA suppresses it^30,34^. We have demonstrated here that flagellin stimulates IL-6 expression in SMCs. In vascular cells, the integration of various activation events induced by flagellin apparently leads to the induction of IL-6 but not of IL-1β or TNF-α. However, the molecular mechanism by which flagellin regulates the differential expression of proinflammatory cytokines in different cell types remains to be elucidated.

Vascular inflammation is known to be mediated by innate immunity molecules, including TLR2 and TLR4, resulting in atherogenesis^5,35^. Inflammation due to innate immunity induces dysfunction of vascular ECs and stimulates the production of proinflammatory cytokines, thereby triggering vascular remodeling, including SMC migration (Fig. 6). Among proinflammatory cytokines, interleukin 6 (IL-6) is a potent regulator coordinating cellular functions to maintain vessel walls^36^. Within the vessel wall, inflammation of vascular SMCs can significantly contribute to vascular remodeling. IL-6 regulates trans-signaling, including SMC-leukocyte interactions. Dysregulated cellular functions resulting from IL-6 production induce SMC migration and deposition of ECM, leading to the development of neointimal hyperplasia in atherosclerosis. Moreover, it has been reported that the TLR4-MyD88 pathway stimulates SMC migration through CREB-dependent IL-6 production^23,37^. In this report, we have shown that the flagellin-TLR5-Nox4 cascade stimulates the expression of proinflammatory cytokines such as RANTES, IL-6 and the adhesion molecule ICAM-1. The chemoattractant RANTES and the adhesion molecule ICAM-1 may be involved in leukocyte recruitment into the intima. Within local inflammation sites, IL-6 serves as a potent component for mediating SMC-leukocyte interactions. Moreover, instability of atherosclerotic plaques occurs predominantly at the fibrous caps of plaques with accumulated inflammatory cells, including macrophages, T-lymphocytes, and VSMCs^38,39^. The accumulated inflammatory cells stimulate the release of inflammatory cytokines, such as IL-6, which is known to be involved in the stimulation of matrix-degrading enzymes. The degradation of the framework by matrix-degrading enzymes triggers instability in the plaque caps, leading to plaque rupture.

Several lines of evidence suggest that flagellin is involved in the production of proinflammatory cytokines, including MIP-2 (CXCL2), RANTES, and IL-6, through the regulation of transcriptional factors, such as AP-1 and NF-kB. RANTES and IL-6 play important roles in the recruitment of vascular SMCa into the intima of the vascular wall to form the fibrous caps of atherosclerotic lesions. Recently, several clinical studies on atherosclerosis have suggested that IL-6 seems to be a potential marker for atherosclerosis. IL-6 levels are associated with future vascular risk^38,40^. A meta-analysis performed by the Emerging Risk Factors Collaboration ultimately demonstrated that an enhanced level of IL-6 is associated with a 25% increase in the risk of future vascular events. IL-6 levels have been shown to correlate with endothelial dysfunction, arterial stiffness, and the extent of subclinical atherosclerosis linked to plaque initiation and destabilization.

In conclusion, we found that atherosclerotic lesion area, necrotic core size, and SMC content were suppressed in Nox4ApoE DKO mice, suggesting a role for Nox4 in neointimal SMC thickening in atherosclerotic plaques. We showed that the TLR4-Nox4-NF-kB signaling cascade stimulates the expression of proinflammatory cytokines and that the TLR4-Nox4-Rac-JNK axis regulates the migration of SMCs. Nox4ApoE DKO mice fed a HFD and injected with rFliC failed to exhibit recruitment of SMCs into the intimal area. Taken together, these results demonstrate that TLR5-mediated Nox4 activation regulates the migration of SMCs, leading to neointimal plaque formation in atherosclerosis.

## Supplementary information

Supplementary Figures
