# Supplementary material for: The flagellin-TLR5-Nox4 axis promotes the migration of smooth muscle cells in atherosclerosis
Source: Exp Mol Med. 2019 Jul 10;51(7):78. doi: 10.1038/s12276-019-0275-6 (PMC6802658; doi:10.1038/s12276-019-0275-6)
Supplement: Supplementary file 1 — Supplementary Figures [file 12276_2019_275_MOESM1_ESM.pptx]

## Slide 1
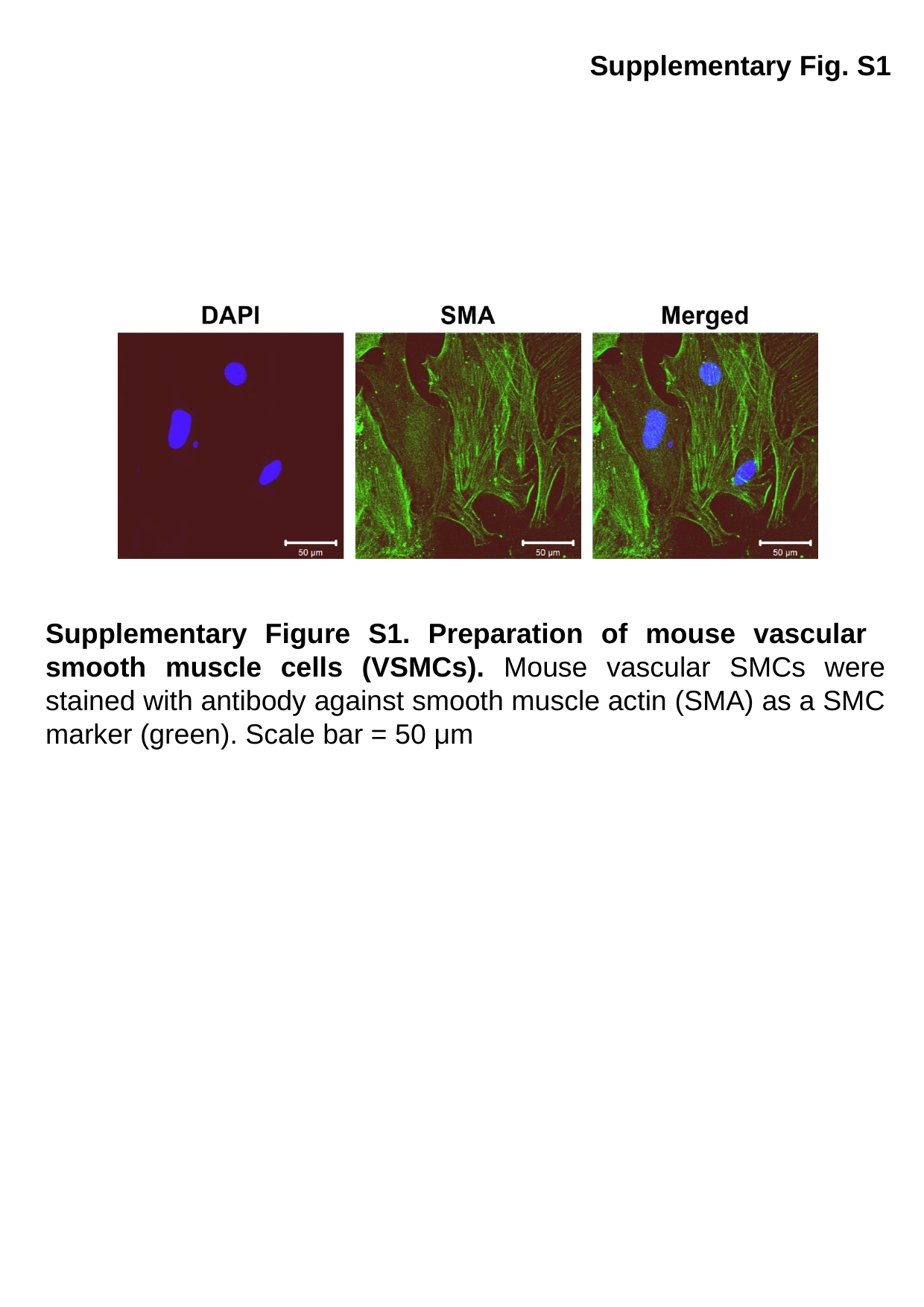

Supplementary Fig. S1
Supplementary Figure S1. Preparation of mouse vascular smooth muscle cells (VSMCs). Mouse vascular SMCs were stained with antibody against smooth muscle actin (SMA) as a SMC marker (green). Scale bar = 50 μm

## Slide 2
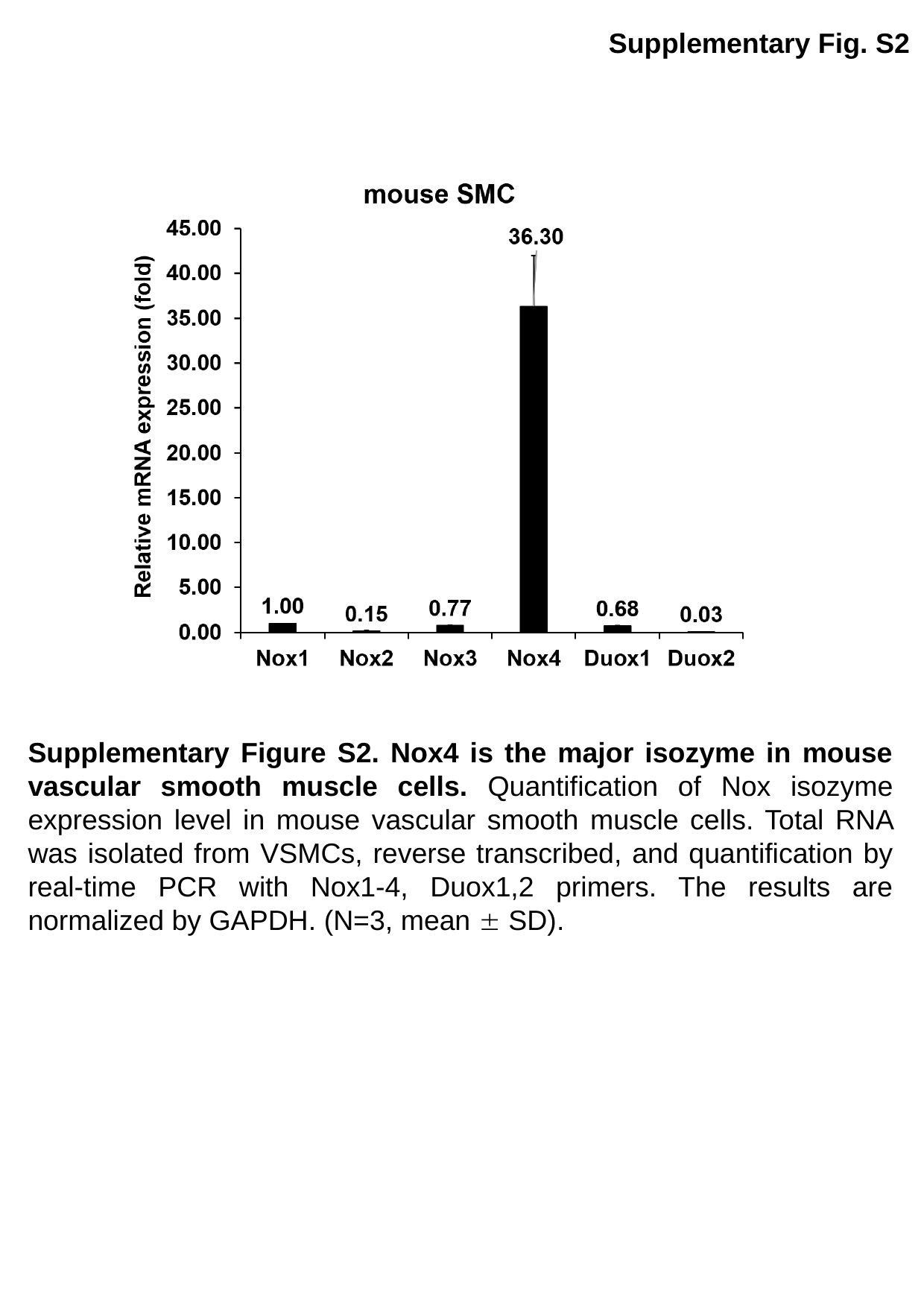

Supplementary Fig. S2
Supplementary Figure S2. Nox4 is the major isozyme in mouse vascular smooth muscle cells. Quantification of Nox isozyme expression level in mouse vascular smooth muscle cells. Total RNA was isolated from VSMCs, reverse transcribed, and quantification by real-time PCR with Nox1-4, Duox1,2 primers. The results are normalized by GAPDH. (N=3, mean ± SD).

## Slide 3
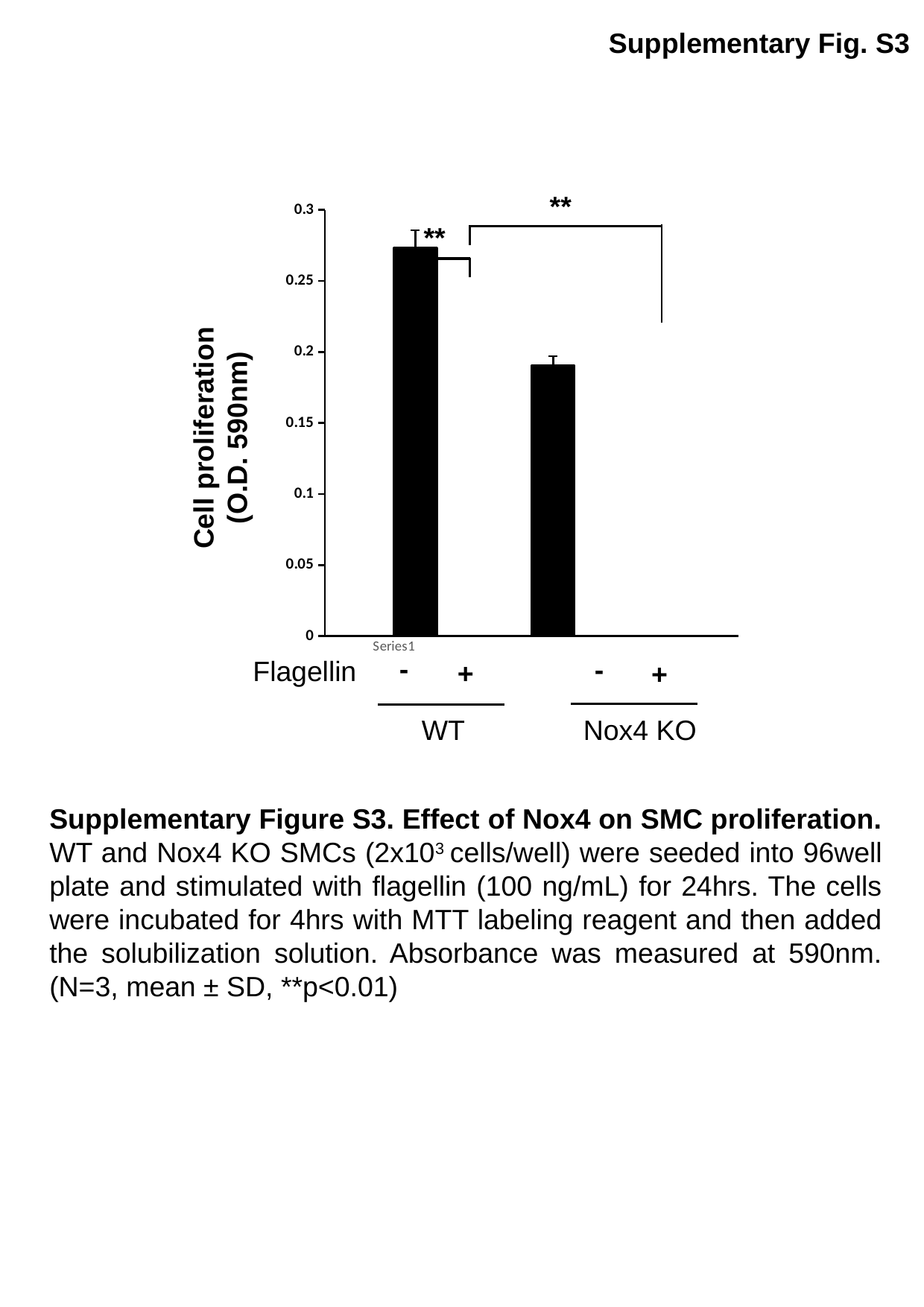

Supplementary Fig. S3
**
### Chart
| Category | | |
|---|---|---|
| | 0.18735000000000002 | 0.27340000000000003 |
| | 0.17801666666666668 | 0.19075 |**
Cell proliferation
(O.D. 590nm)
-
-
Flagellin
+
+
WT
Nox4 KO
Supplementary Figure S3. Effect of Nox4 on SMC proliferation. WT and Nox4 KO SMCs (2x10^3^ cells/well) were seeded into 96well plate and stimulated with flagellin (100 ng/mL) for 24hrs. The cells were incubated for 4hrs with MTT labeling reagent and then added the solubilization solution. Absorbance was measured at 590nm. (N=3, mean ± SD, **p<0.01)

## Slide 4
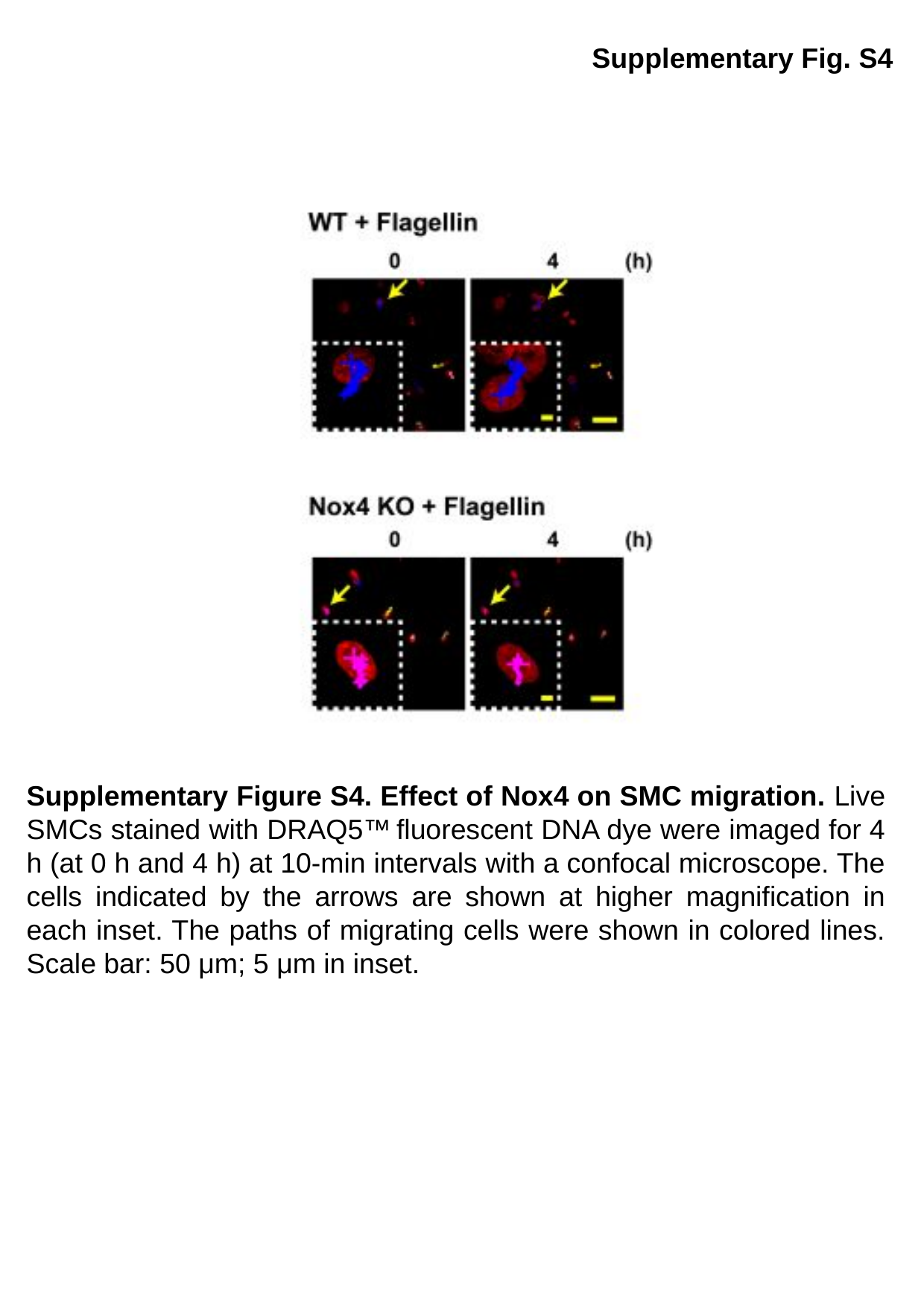

Supplementary Fig. S4
Supplementary Figure S4. Effect of Nox4 on SMC migration. Live SMCs stained with DRAQ5™ fluorescent DNA dye were imaged for 4 h (at 0 h and 4 h) at 10-min intervals with a confocal microscope. The cells indicated by the arrows are shown at higher magnification in each inset. The paths of migrating cells were shown in colored lines. Scale bar: 50 μm; 5 μm in inset.

## Slide 5
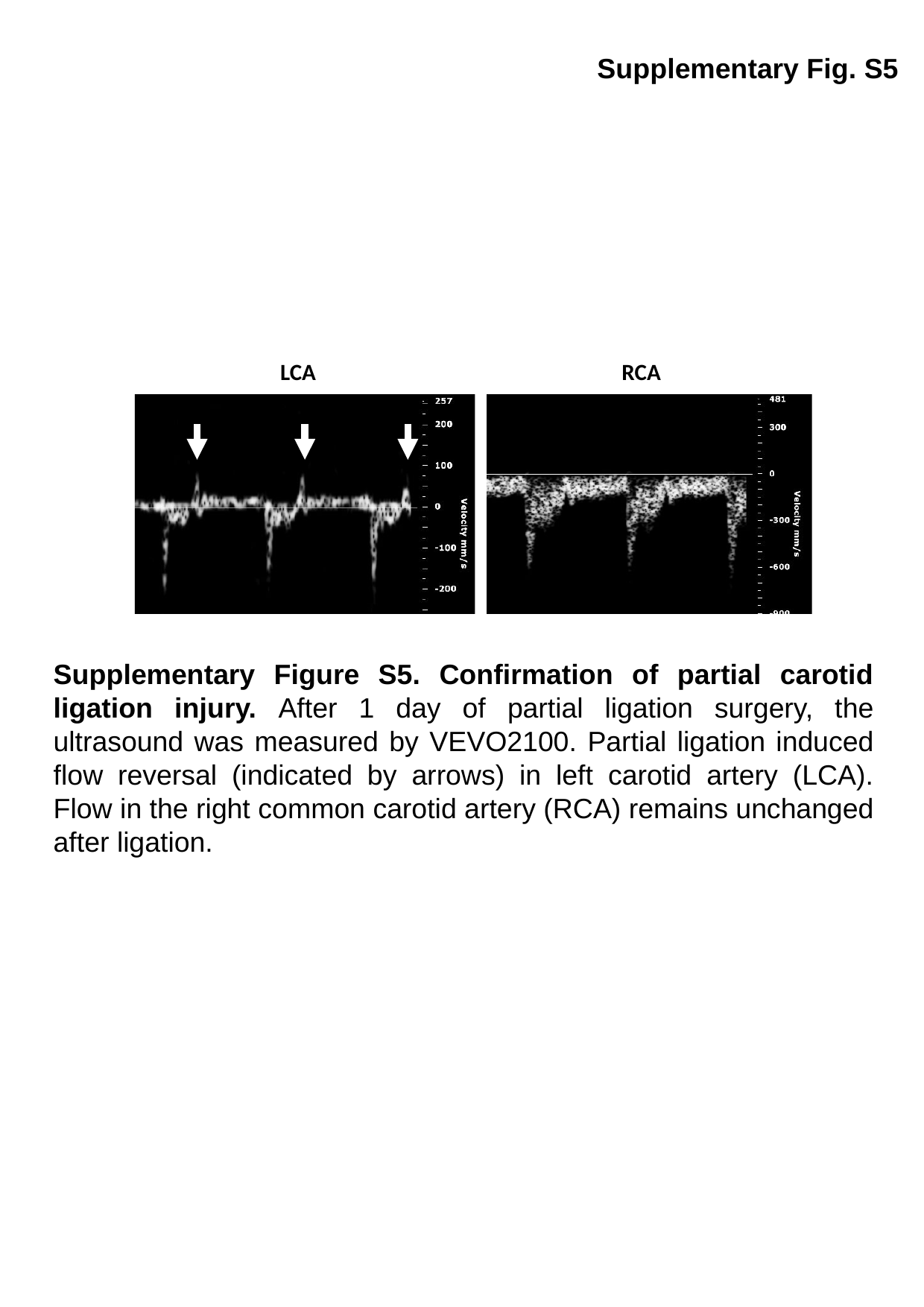

Supplementary Fig. S5
LCA
RCA
Supplementary Figure S5. Confirmation of partial carotid ligation injury. After 1 day of partial ligation surgery, the ultrasound was measured by VEVO2100. Partial ligation induced flow reversal (indicated by arrows) in left carotid artery (LCA). Flow in the right common carotid artery (RCA) remains unchanged after ligation.

## Slide 6
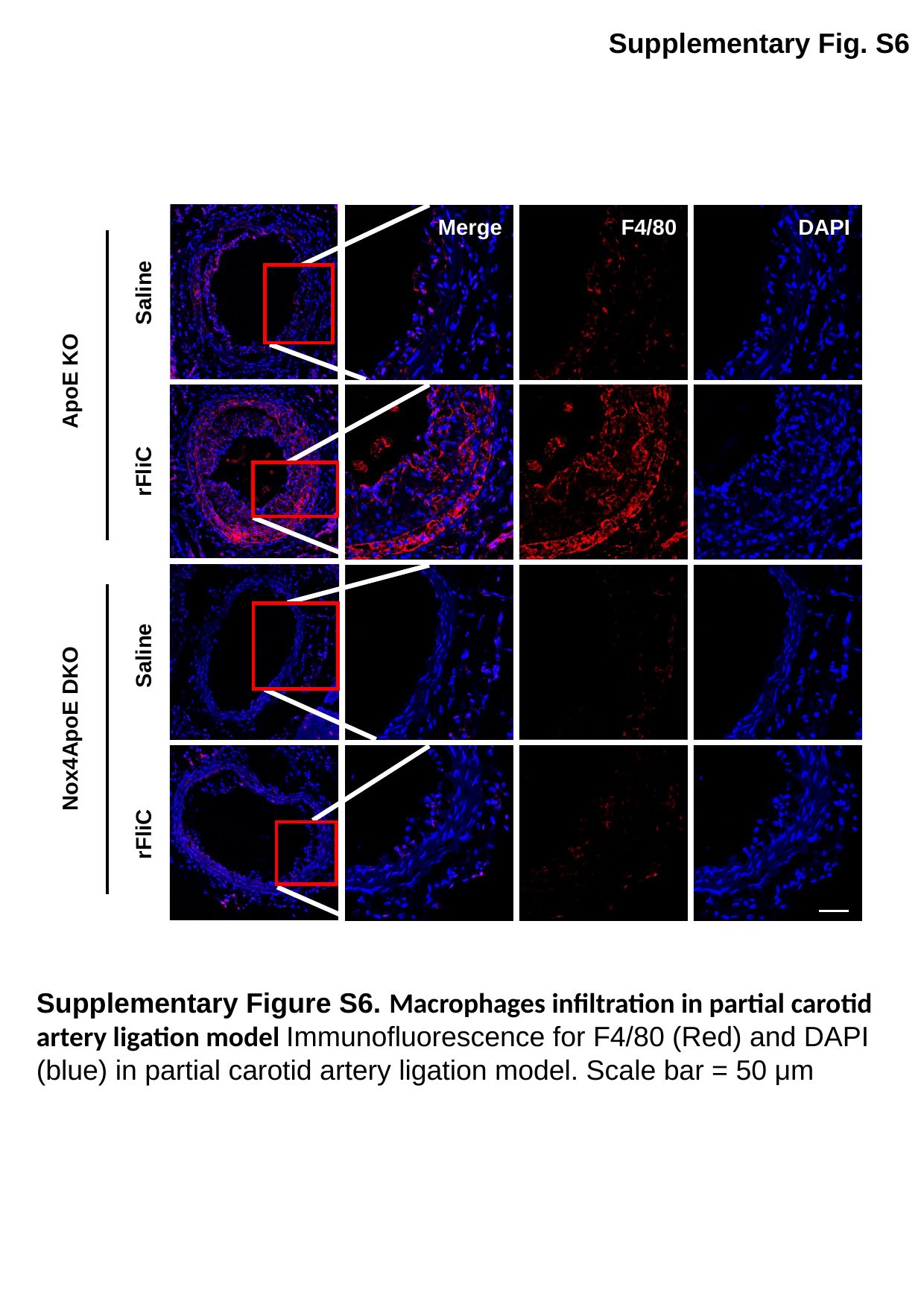

Supplementary Fig. S6
Merge
F4/80
DAPI
Saline
ApoE KO
rFliC
Saline
Nox4ApoE DKO
rFliC
Supplementary Figure S6. Macrophages infiltration in partial carotid artery ligation model Immunofluorescence for F4/80 (Red) and DAPI (blue) in partial carotid artery ligation model. Scale bar = 50 μm
